# Methodological and Statistical Confounding in Comparing Patient‐Reported Outcomes Between Cardioversion and Ablation for Atrial Fibrillation

**DOI:** 10.1002/clc.70407

**Published:** 2026-07-04

**Authors:** Faizan Raza

**Affiliations:** ^1^ Continental Medical College Lahore Pakistan

To the Editor,

1

I read with great interest the article by Oren et al. [1] evaluating patient‐reported outcome measures (PROMs) following elective electrical cardioversion versus catheter ablation for atrial fibrillation. While I commend the authors for investigating clinically relevant endpoints like sleep, health, and anxiety, several structural inconsistencies and methodological confounding factors limit the finality of their conclusions.

## 2 Methodological Concerns and Structural Inconsistencies

### 2.1 Data Contradictions in Sample Size

A prominent mathematical contradiction exists regarding participant enrollment. In section 2.5, the authors stated that the study targeted 30 participants per group (*n* = 60) and that due to one dropout in each group, the study was completed with 58 participants. However, the abstract and result explicitly state that the study included 27 patients in the ablation group and 30 in the cardioversion group, totaling 57 patients. If exactly one dropout occurs in each group, the ablation cohort should consist of 29 patients, not 27. This discrepancy leaves two missing or unaccounted‐for data profiles in the ablation arm, raising concerns regarding data transparency and potential attrition bias.

### 2.2 Unadjusted Baseline Selection Bias

While baseline sociodemographics were homogeneous in Table 1, Table 3 reveals that the two cohorts were highly unequal psychometrically before any interventions took place. At baseline, the ablation group suffered from significantly lower quality of life (AFEQT: 40.33 ± 21.75 vs. 57.50 ± 16.13), higher anxiety (BAI35.81 ± 9.42 vs. 30.07 ± 6.25), and worse sleep quality (PSQI 11.19 ± 4.54 vs. 8.73 ± 4.37). In non‐randomized quasi‐experimental designs, this indicates a strong selection bias where physicians naturally channel sicker, highly symptomatic patients toward the more invasive ablation pathway.

Comparing the absolute post‐treatment course using the WELCH'S test overlooks this profound imbalance. Notably, the ablation achieved a much larger absolute improvement in quality of life (+21.19 points) compared to the cardioversion group (+10.86). By focusing primarily on post‐treatment absolute scores to conclude that cardioversions yields greater reductions in anxiety and sleep disturbances the authors interpretation inadvertently penalizes the sicker ablation cohort. An analysis of covariance (ANCOVA) should have been utilized to statistically adjust for their baseline discrepancies [2].

### 2.3 Inadequate Follow‐up and Blanking Period Confounding

The 30–45 day follow‐up window truncates the universally recognized 3‐month (90 days) blanking period typical in ablation trials, meaning data collection concluded prematurely while transient post‐procedural inflammation and early recurrences were still clinically active. The first 3 months post‐ablation are characterized by transient early arrhythmias, groin access discomfort, and myocardial inflammation. Expecting an ablation patient to display fully resolved sleep or anxiety patterns at merely 30 days is clinically unrealistic. The statistically nonsignificant change in sleep quality (*p* = 0.092) observed in the ablation group likely reflects early postoperative physical recovery rather than a limitation of the intervention itself. Additionally, collecting post‐data via telephone for a subset of patients introduces a mode of administration bias [3].

## 3 Conclusion and Future Recommendations

To enhance future comparison of rhythm control strategies, the investigator should implement a minimum of 6−12 months follow‐up timeline to outlast the blanking period. Furthermore, utilizing ANCOVA modeling is essential to properly account for pre‐existing baseline psychometric variations.

## Data Availability

Data sharing is not applicable to this article as no data sets were generated or analyzed during the current study.

## References

[1] B. Oren , B. Z. Buyukkilic , K. Soydas , and E. Oren , “A Comparative Analysis of the Effects of Cardioversion and Ablation on Anxiety, Sleep, and Quality of Life in Patients Diagnosed With Atrial Fibrillation.” Clinical Cardiology 49, no. 6 (Wiley Online Library, 2026), e25450. 10.1002/clc.70388 PMC13288365 42334044

[2] A. J. Vickers and D. G. Altman , “Statistics Notes: Analysing Controlled Trials With Baseline and Follow up Measurements.” British Medical Journal 323, no. 7321 (2001): 1123–1124. 11701584 10.1136/bmj.323.7321.1123 PMC1121605

[3] H. Calkins , G. Hindricks , R. Cappato , et al. “HRS/EHRA/ECAS/APHRS/SOLAECE Expert Consensus Statement on Catheter and Surgical Ablation of Atrial Fibrillation.” Heart Rhythm 14, no. 10 (EP Europace Oxford Academic, 2017), e275–e444. 28506916 10.1016/j.hrthm.2017.05.012 PMC6019327

